# Astrocyte in Neurological Disease: Pathogenesis and Therapy

**DOI:** 10.1002/mco2.70299

**Published:** 2025-07-17

**Authors:** Meihui Huang, Aoyang Long, Lingjia Hao, Zilin Shi, Mengqi Zhang

**Affiliations:** ^1^ Department of Neurology Xiangya Hospital, Central South University Changsha Hunan China; ^2^ National Clinical Research Center for Geriatric Disorders Xiangya Hospital, Central South University Changsha Hunan China

**Keywords:** astrocyte, blood–brain barrier dysfunction, metabolic reprogramming, neuroinflammation, neurological disease, Therapeutic strategies

## Abstract

Astrocytes, as key support cells in the central nervous system, maintain homeostasis in the brain through mechanisms such as ionic homeostasis regulation, metabolic support, synaptic modulation, and neuroinflammatory regulation. Recent studies have shown that astrocyte dysfunction is closely related to the pathologic processes of various neurological diseases, such as Alzheimer's disease, Parkinson's disease, multiple sclerosis, and stroke. In this review, we comprehensively summarize the dual roles of astrocytes in neurological diseases: on one hand, their aberrant activation can exacerbate disease progression by mediating neuroinflammation, synaptic dysfunction, and blood–brain barrier disruption; on the other hand, their metabolic disorders, such as lipid droplet accumulation, mitochondrial dysfunction, and oxidative stress imbalance, further drive neurodegeneration. In terms of therapeutic strategies, interventions targeting astrocytes, such as modulation of activation phenotype, metabolic reprogramming, gene therapy, and innovative therapies based on exosomes and nanotechnology, show great promise. In the future, we may integrate multi‐omics technologies and deepen clinical translational research to systematically analyze the spatial heterogeneity of astrocytes and their dynamic regulatory networks at different stages of the disease, in order to elucidate the precise mechanisms of their roles in the pathological process, and thus provide multidimensional theoretical support for the design of targeted therapeutic strategies.

## Graphical abstract

This review provides comprehensive overview of astrocytes in neurological disorders. Astrocytes contribute to neurological disorders via A1/A2 polarization, inducing neuroinflammation, synaptic, and BBB dysfunction. Metabolic disturbances in glucose, lipid, and amino acid pathways leading to oxidative stress and mitochondrial damage. Therapeutic targets include inflammation modulation, gene therapy, and nanotechnology. Future directions include multi‐omics integration, clinical translation, and resolving astrocyte heterogeneity.

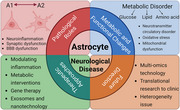

## 1 Introduction

The central nervous system (CNS) is the core structure that regulates the complex physiological functions of the body. Its normal operation depends on the close cooperation of various cell types. Among these cells, astrocytes, as the predominant glial cell type in the CNS, play a critical role in maintaining ionic homeostasis, providing metabolic support, regulating synaptic function, and other essential processes [1]. Furthermore, astrocytes are indispensable for the survival and functional integrity of neurons through their provision of trophic factors, clearance of extracellular debris, and maintenance of redox balance [2]. However, the role of astrocytes is not always beneficial. Under healthy conditions, their supportive functions are crucial, but under pathological conditions, they can transform into pro‐inflammatory factors that contribute to neurological diseases [3, 4].

Under normal physiological conditions, astrocytes have multifaceted roles that support the smooth operation of the CNS. First, they regulate the concentrations of potassium and calcium ions, maintaining the excitability balance of neurons and thus playing a key role in ionic homeostasis [5]. Second, astrocytes regulate lactate and glucose metabolism, providing energy support for neurons and ensuring efficient neuronal activity [6]. Third, astrocytes actively participate in neurotransmitter recycling, particularly through the glutamate–glutamine cycle, which is essential for preventing excitotoxicity and maintaining proper synaptic signaling [7]. Moreover, astrocytes protect neurons from external stress and injury by secreting neurotrophic factors and modulating inflammatory responses [2, 8]. However, the complexity of these functions means that any disruption in astrocyte function can become a pivotal starting point for various CNS pathological changes, thereby contributing to the onset and progression of neurological diseases [9].

In multiple disease conditions, astrocytes shift from a protective to a damaging role, with their reactive state often regarded as one of the core mechanisms of pathological processes. For instance, in Alzheimer's disease (AD), astrocytes respond to the pathological deposition of β‐amyloid (Aβ), releasing pro‐inflammatory factors that exacerbate neuroinflammation, thus promoting disease progression [10]. In Parkinson's disease (PD), astrocytes are closely associated with the degeneration of dopaminergic neurons, and their metabolic dysfunction and inflammatory responses may be key drivers of neuronal death [11, 12]. In multiple sclerosis (MS), reactive astrocytes contribute to demyelination through the production of chondroitin sulfate proteoglycans and impede oligodendrocyte precursor differentiation, ultimately hindering remyelination processes [13]. On the other hand, Ji et al. demonstrate the tumor‐promoting role of senescent astrocytes in the irradiated glioma microenvironment [14]. During the pathological process of stroke, astrocytes display a dual role: they can promote neural recovery by repairing barriers and reducing inflammation, but they may also exacerbate damage through excessive inflammation and metabolic dysregulation [15, 16, 17]. In conclusion, the multifaceted and reactive characteristics of astrocytes in various diseases make them a key pathological mechanism in CNS disorders, warranting in‐depth study.

Given the critical role of astrocytes in CNS diseases, therapeutic strategies targeting their function have received increasing attention in recent years [18]. Current research highlights the potential of targeting astrocytes in multiple ways [19]. For example, small molecule drugs can alleviate pathological damage by modulating their inflammatory responses [20]. Gene therapies can intervene in diseases at their source by repairing gene expression dysregulation related to astrocyte function [21]. Moreover, neuroprotective agents can restore the normal function of astrocytes, providing supportive effects for damaged neural environments [22]. Recent preclinical investigations and emerging clinical trials have demonstrated the therapeutic potential of pharmacological interventions targeting astrocytes, including anti‐inflammatory compounds, metabolic modulators, and agents that promote beneficial astrocytic phenotypes [23]. However, despite the promising potential of these research directions, astrocyte‐targeted therapies still face many challenges, including issues related to specificity and side effects. Future research needs to explore how to more precisely regulate astrocyte function, thereby developing safe and effective therapeutic strategies.

**FIGURE 1:**
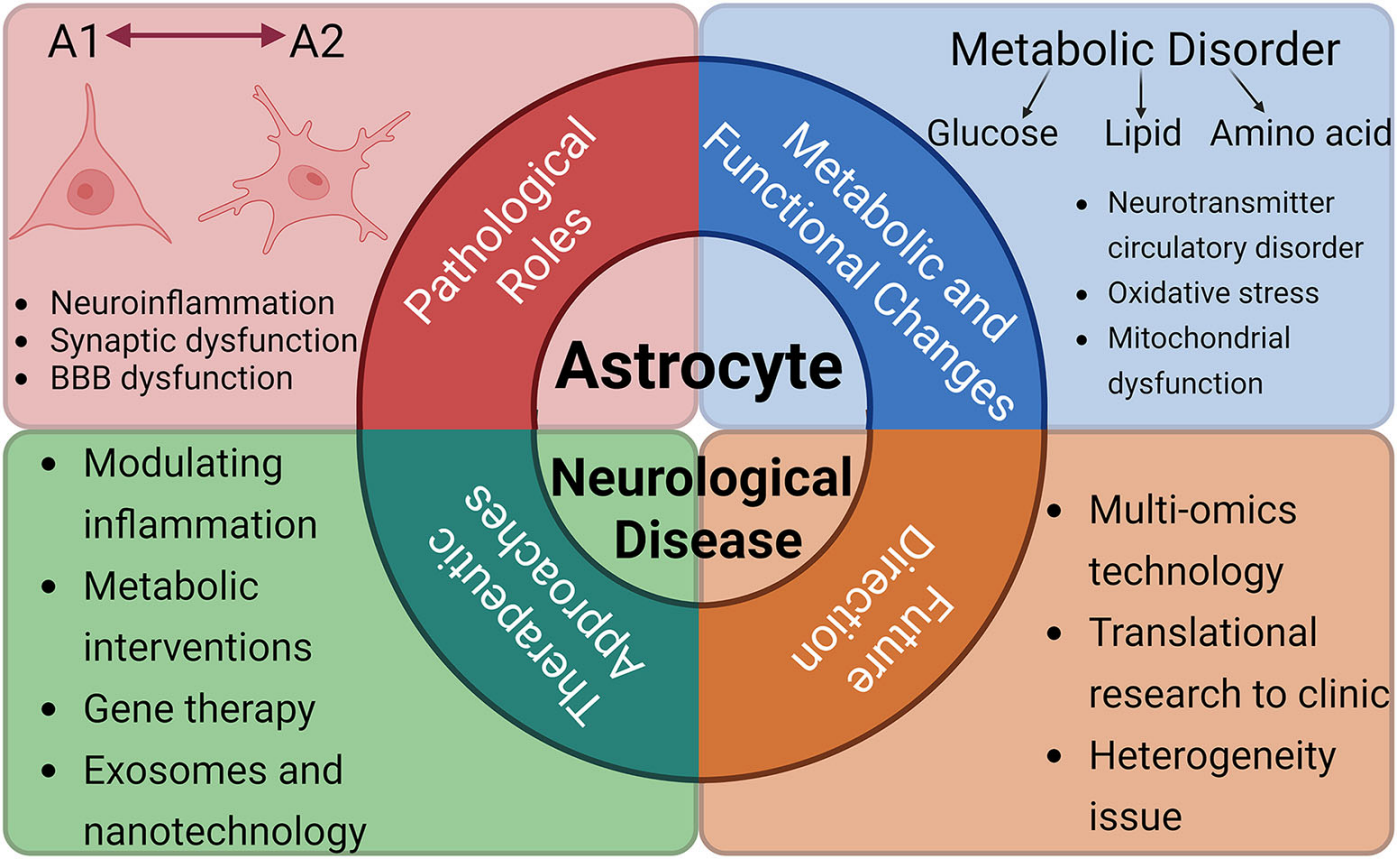
Astrocytes in neurological diseases. Astrocytes contribute to neurological disorders via A1/A2 polarization, inducing neuroinflammation, synaptic, and BBB dysfunction. Metabolic disturbances in glucose, lipid, and amino acid pathways leading to oxidative stress and mitochondrial damage. Therapeutic targets include inflammation modulation, gene therapy, and nanotechnology. Future directions include multi‐omics integration, clinical translation, and resolving astrocyte heterogeneity.

This article aims to systematically summarize the roles of astrocytes in CNS diseases, their pathological mechanisms, and the existing targeted therapeutic strategies, while proposing potential directions for future research. By doing so, we hope to provide a theoretical foundation and practical insights for understanding the pathological processes of CNS diseases and developing innovative therapies (Figure 1).

## 2 The Pathological Role of Astrocytes in Nervous System Diseases

### 2.1 Neuroinflammation and Reactive Astrocytes

In the field of neuroscience, neuroinflammation, as a common pathological process and neurological symptom, is involved in the pathogenesis of many nervous system diseases, such as AD and depression [24, 25]. Its complexity and diversity profoundly affects the homeostasis and normal function of the nervous system. Neuroinflammation can be triggered by a variety of factors, including infectious agent invasion, traumatic brain injury (TBI), neurodegenerative process, and autoimmune disorders. These factors break the originally precisely regulated internal environment balance of the CNS and start a series of complex inflammatory cascades.

As one of the most abundant and functionally critical glial cell types in the CNS, astrocytes play an important role in the occurrence and development of neuroinflammation. Experimental evidence demonstrates that lipopolysaccharide (LPS) administration in rats upregulates toll‐like receptor 4 (TLR4) expression, which subsequently activates downstream signaling cascades including protein kinase pathways, extracellular signal‐regulated kinase (ERK1/2), and nuclear factor kappa B (NF‐κB) transcription factors, ultimately inducing the expression of pro‐inflammatory cytokines such as interleukin‐1 (IL‐1). In the process of neuroinflammation, the content of astrocyte marker glial fibrillary acidic protein (GFAP) increased, reflecting the close relationship between astrocyte response and neuroinflammatory pathway activation [26].

#### 2.1.1 Reactive Astrocytes Are Involved in the Mechanism of Neuroinflammation

As important immunocompetent glial cells are in the CNS, when the homeostasis of the brain is disrupted, astrocytes can undergo conformational changes to transform into reactive astrocytes. Compared with normal astrocytes, the latter can show morphological changes such as cell hypertrophy, reduced number and swelling of processes, and enlarged nuclei. Related changes in astrocytes have been observed in the LPS‐induced neuroinflammation model in mice [27, 28].

In neuroinflammation, reactive astrocytes exhibit a dichotomous functional profile. On one hand, they can restrict inflammatory spread through formation of glial scars, secrete neurotrophic factors that support neuronal repair, upregulate glutamate transporters to mitigate excitotoxicity, and engage in multiple mechanisms that attenuate neuroinflammatory damage to the CNS. On the other hand, they can produce inflammation‐related cytokines such as IL‐6, IL‐1β, reactive oxygen species (ROS), inflammatory cell chemokines, leading to aggravation of inflammation, inhibition of axon regeneration, and neuronal damage [29, 30, 31]. For example, Hong et al. pointed out that ciliary neurotrophic factor (CNTF) can act on astrocytes through the IL‐6/IL‐6R pathway to induce the release of inflammatory related cytokines IL‐6, tumor necrosis factor (TNF)‐α, and IL‐1β, thereby promoting neuroinflammation. This increased IL‐6 signaling subsequently leads to intracellular calcium dysregulation in neurons, triggering ROS production and exacerbating neuroinflammatory processes. In addition, cytokines produced by astrocytes can interact with each other in neuroinflammation through the cytokine network, and further activate astrocytes to release more cytokines and aggravate inflammatory response [32, 33].

#### 2.1.2 Two Subtypes of Astrocytes in Neuroinflammation

**FIGURE 2:**
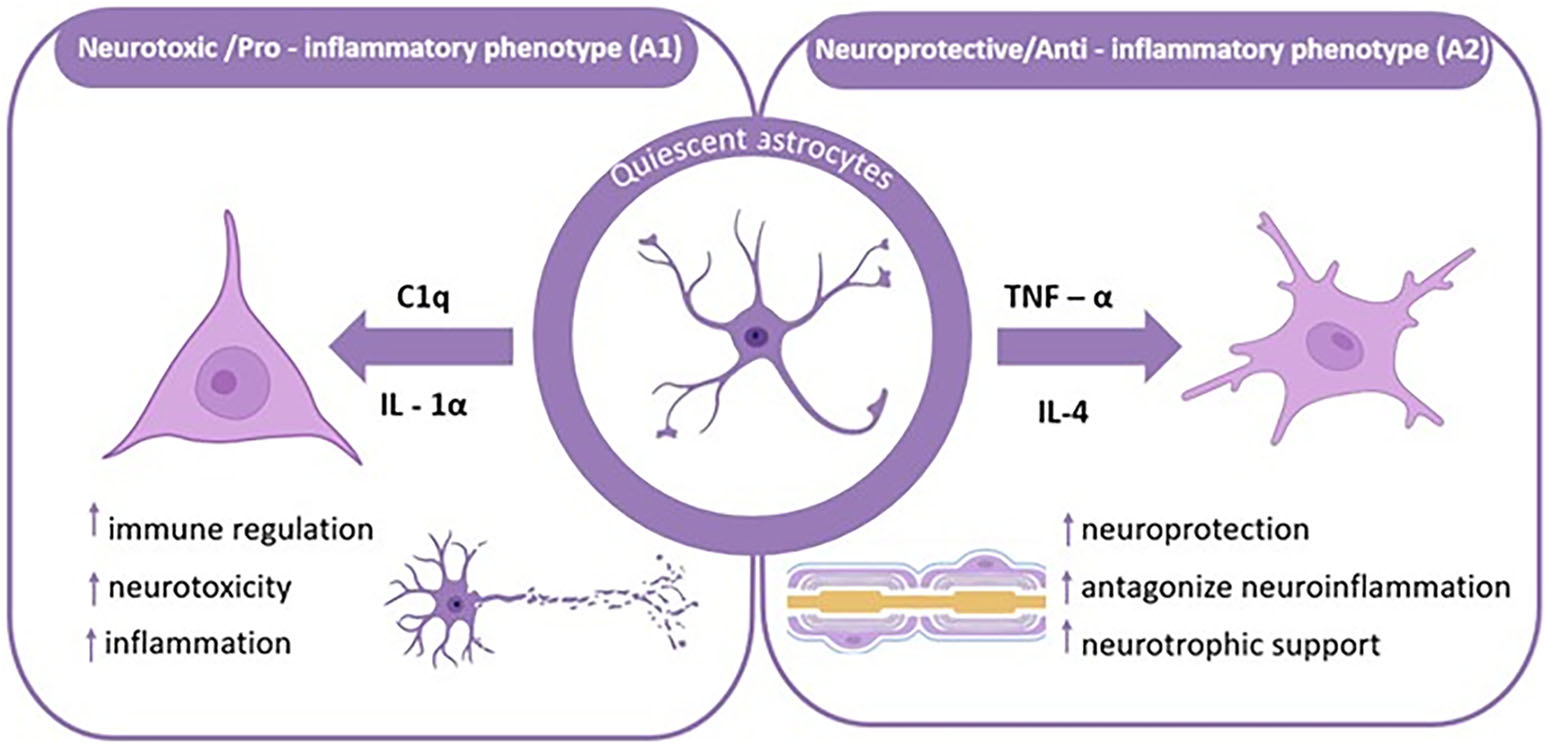
Schematic illustration of the differentiation of quiescent astrocytes into two distinct phenotypes. In the presence of C‐1q and IL‐1α, quiescent astrocytes transform into the neurotoxic/pro‐inflammatory phenotype (A1), which is associated with immune regulation, neurotoxicity, and inflammation. Conversely, under the influence of TNF‐α and IL‐4, they differentiate into the neuroprotective/anti‐inflammatory phenotype (A2), exhibiting functions such as neuroprotection, antagonizing neuroinflammation, and providing neurotrophic support.

In response to neuroinflammatory stimuli, astrocytes can polarize into two distinct functional phenotypes: the neurotoxic or pro‐inflammatory phenotype (A1) and the neuroprotective or anti‐inflammatory phenotype (A2), as illustrated in Figure 2. During LPS‐induced neuroinflammation, A2 subtype gene expression was found to be correlated with A1 subtype gene expression. They are involved in the regulation of astrocytes in inflammation [34]. The A1 subtype is mainly produced by activated microglia to induce astrocytes. Upon activation, the former can release mediators such as IL‐1α, TNF‐α, and complement component 1q (C‐1q) to polarize astrocytes into A1 phenotype [35]. In studies related to spinal cord injury, it has been found that A1 subtype can release soluble toxins, destroy CNS related neurons and microglia, and induce synapse formation dysfunction, which is neurotoxic and pro‐inflammatory to a certain extent [36].

In contrast to the neurotoxic A1 phenotype, A2 astrocytes exert neuroprotective functions through the upregulation of neurotrophic factors, anti‐inflammatory cytokines, and TGF‐β, thereby supporting neuronal survival, promoting synaptogenesis, and mitigating neuroinflammatory processes [31, 37]. At the same time, the A2 marker S100A10 protein plays an important role in neuronutrition and support, intracellular transport, and cell migration. Experimental investigations utilizing astrocyte‐specific connexin‐30 (Cx30) knockout (KO) mice treated with 1‐methyl‐4‐phenyl‐1,2,3,6‐tetrahydropyridine (MPTP) demonstrated that S100a10 expression is significantly upregulated on the first day following MPTP administration in both wild‐type (WT) and Cx30 KO mice, suggesting that S100a10 induction represents an early adaptive response to acute neurotoxic injury [36, 38, 39].

### 2.2 Synaptic Dysfunction and Neurotransmitter Imbalance

As the most widely distributed glial cells in the CNS, astrocytes have a wide range of information regulation functions. These cells not only sense and respond to synaptic neurotransmitter release but also actively participate in modulating synaptic formation, maturation, and function through various mechanisms, including the establishment of tripartite synapses [40].

#### 2.2.1 Normal Regulation of Synaptic Function and Neurotransmitter Balance by Astrocytes

Astrocytes serve as fundamental architects of synaptic connectivity and neurotransmitter homeostasis in the CNS, orchestrating multiple interconnected processes that ensure optimal neural circuit function [41]. During synaptogenesis, astrocytes orchestrate the formation of excitatory synapses by secreting synaptogenic factors such as thrombospondins (TSPs) and glypicans 4/6, which bind to neuronal surface receptors and initiate synapse assembly. In addition, astrocytes secrete synaptogenic proteins such as neurocan to regulate the formation of inhibitory synapses by cleaving neuroglycans [42, 43]. For developmental refinement of neural circuits, astrocytes mediate synapse elimination and pruning through the expression of phagocytic receptors including MEGF10 and MERTK, which recognize and engulf synaptic components targeted for removal [42, 44, 45]. The “tripartite synapse” usually refers to the complex interaction and regulation between astrocytes, presynaptic neurons, and postsynaptic neurons. It is mainly composed of presynaptic neuron terminals, postsynaptic neurons, and astrocyte processes [46]. This conceptual framework represents a paradigm shift from conventional neuron‐centric views of synaptic transmission to one that acknowledges the integral role of astrocytes in information processing and synaptic plasticity. Neurons release neurotransmitters such as glutamate and gama‐aminobutyric acid (GABA) that not only act on their neuronal targets but also bind to receptors on perisynaptic astrocytes, triggering intracellular calcium signaling cascades. These calcium fluctuations within astrocytes can, in turn, modulate the release of gliotransmitters that regulate synaptic activity across astrocytic networks [47, 48].

**FIGURE 3:**
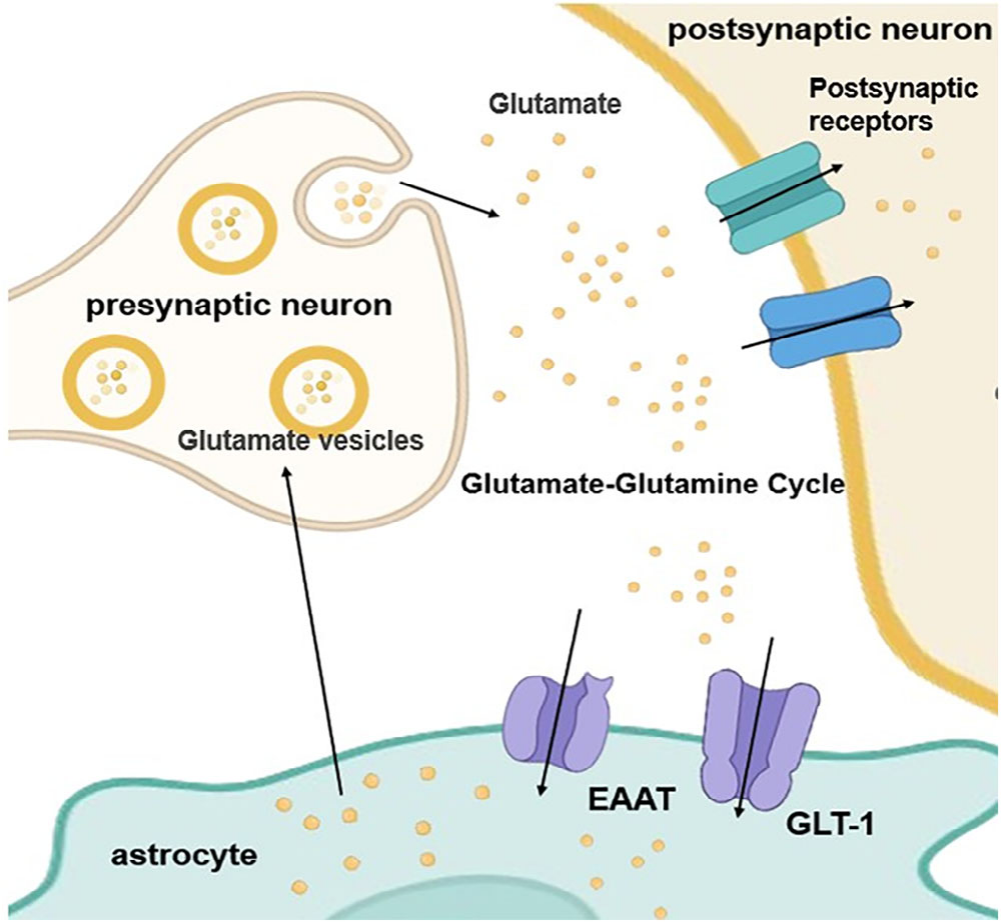
Schematic diagram of the glutamate–glutamine cycle at the synapse. The presynaptic neuron releases glutamate into the synaptic cleft. Glutamate can bind to receptors on the postsynaptic neuron. Meanwhile, the astrocyte utilizes excitatory amino acid transporters (EAAT) and GLT‐1 transporters to take up glutamate from the synaptic cleft and convert it into glutamine. This cycle plays a crucial role in maintaining proper synaptic function and glutamate homeostasis.

Astrocytes are pivotal contributors to neurotransmitter metabolism and cycling in the brain. For example, in the glutamate–glutamine cycle (Figure 3), cytosolic glutamate is first stored in synaptic vesicles through ATP hydrolysis by vesicular glutamate transporter (VGLUT) and chloride ions [49, 50]. Subsequently, glutamate is released into the synaptic cleft through exocytosis and activates glutamate receptors in the postsynaptic membrane, thereby mediating excitatory signal transmission. Following synaptic activity, extracellular glutamate must be rapidly cleared to prevent excitotoxicity. This clearance is primarily accomplished through high‐affinity glutamate transporters, including GLT‐1 and GLAST (predominantly expressed in astrocytes) and excitatory amino‐acid transporters 3 (EAAT3), EAAT4, and EAAT5 (mainly expressed in neurons) [7]. Neurons and astrocytes can take up glutamate from the synaptic cleft through relevant transporters. The latter (astrocytes) can convert most of the ingested glutamate into glutamine through glutamine synthetase (GS). The resulting glutamine is subsequently released into the extracellular space, where it is taken up by neurons and reconverted to glutamate by phosphate‐activated glutaminase (PAG), thus completing the intercellular neurotransmitter recycling pathway [9, 51, 52, 53].

#### 2.2.2 Dysregulation of Synaptic Function and Neurotransmitter Imbalance Caused by Astrocyte Dysfunction

Pathological disruption of astrocyte‐mediated synaptic regulation and neurotransmitter homeostasis represents a critical convergence point in the pathogenesis of diverse neurological disorders. In AD, the pathological features mainly include Aβ deposition and neuronal tangles (NFT) formed by tau protein. Subsequent investigations have confirmed this hypothesis, demonstrating significantly reduced expression of the astrocytic glutamate transporter GLT‐1 in the vicinity of amyloid plaques, which impairs glutamate clearance and contributes to excitotoxicity [54, 55, 56]. Furthermore, in transgenic mouse models of AD, reactive astrocytes surrounding amyloid deposits have been shown to release excessive GABA through the Ca^2^⁺‐activated anion channel bestrophin 1 (BEST1), with this aberrant GABA release being mediated by upregulated monoamine oxidase B (MAOB). The resulting tonic inhibition disrupts synaptic plasticity and contributes to cognitive impairment characteristic of AD pathology [57]. In PD, astrocytes can affect the balance of glutamate neurotransmitters, participate in glutamate‐mediated excitotoxicity, and other processes, affect the survival of dopaminergic neurons, interfere with the synaptic transmission of dopaminergic neurons, and then affect the function of CNS neural circuit, leading to related symptoms [9]. Collectively, these findings demonstrate that astrocyte dysfunction leads to neurotransmitter imbalance and synaptic abnormalities that play pivotal roles in the pathogenesis and progression of diverse neurological disorders.

### 2.3 Astrocyte‐Mediated Blood–Brain Barrier Dysfunction

The blood–brain barrier (BBB) represents a highly specialized interface between the circulatory system and the CNS, comprising cerebrovascular endothelial cells interconnected by tight junctions, along with pericytes, basement membrane, and astrocytic end‐feet processes. This neurovascular complex not only regulates blood flow and selective exchange of nutrients and signaling molecules but also provides critical neuroprotection through its physical barrier function [58]. Among these components, astrocytes play a particularly pivotal role in BBB homeostasis through their unique anatomical and functional relationships with cerebral vasculature.

Astrocytic end‐feet processes form extensive perivascular coverage around cerebral blood vessels, creating a nearly continuous anatomical interface that maintains barrier integrity through complementary mechanisms. These specialized structures provide physical protection, shielding the CNS from exogenous and endogenous toxins while actively facilitating the transport of essential nutrients and metabolic precursors [59, 60]. The homeostatic functions of astrocytes are mediated through secreted factors including sonic hedgehog (Shh), retinoic acid, and angiopoietin‐1, which collectively enhance BBB integrity by upregulating endothelial tight junction proteins [61, 62]. However, this delicate balance can be disrupted under pathological conditions, where astrocyte dysfunction frequently initiates BBB breakdown—a hallmark of numerous CNS disorders.

Notably, astrocyte‐derived apolipoprotein E (APOE) exemplifies the dual roles of astrocytic signaling in BBB regulation. Primarily synthesized by astrocytes with minor contributions from microglia and oligodendrocytes, APOE serves critical functions in lipid transport and neural maintenance [63, 64]. Genetic studies using astrocyte‐specific APOE manipulations reveal that isoform‐specific effects significantly impact vascular permeability: APOE4 (the major genetic risk factor for AD) compromises BBB integrity through cyclophilin A (CypA)‐mediated signaling cascades, while simultaneously reducing astrocytic end‐foot coverage of blood vessels [65, 66]. This APOE4‐dependent pathway provides a direct molecular link between astrocytic dysfunction and neurodegenerative disease progression.

Under neuroinflammatory conditions, reactive astrocytes undergo profound functional shifts that exacerbate BBB damage. Pathological stimuli including infections, trauma, or neurodegeneration trigger astrocyte activation and subsequent release of permeability‐altering factors. Three major mechanisms have been characterized: First, astrocytes secrete vascular endothelial growth factor A (VEGFA) and thymidine phosphorylase (TYMP), which directly increase endothelial permeability [67, 68]. Second, their production of pro‐inflammatory cytokines such as IL‐1 disrupts tight junction complexes [69, 70]. In addition, activated astrocytes initiate inflammatory signaling cascades involving the signal transducer and activator of transcription 3 (STAT3) and mitogen‐activated protein kinase/extracellular signal‐regulated kinase (MEK/ERK) pathways.

Transcriptomic studies demonstrate that cytokines like IL‐1β and TNF‐α induce STAT3‐mediated overexpression of α1‐antitrypsin (α1ACT) in astrocytes, creating a self‐reinforcing cycle of BBB dysfunction [71]. In PD models, this process is further amplified by leucine‐rich repeat kinase 2 (LRRK2) G2019S mutations in astrocytes, which enhance MEK/ERK activation and elevate secretion of IL‐6 and IL‐8, ultimately accelerating disease progression [72].

## 3 Metabolic and Functional Changes in Astrocytes in Neurological Diseases

Astrocytes are the most numerous and widely distributed glial cell population in the CNS. It performs key physiological processes such as BBB formation and maintenance, synaptogenesis, neurotransmission, and metabolic regulation, and plays a central role in maintaining brain homeostasis [73]. Particularly important is that the precise balance of metabolic pathways in this cell population, as the main regulator of brain energy metabolism, is essential for the normal operation of neural functions. Recent studies have shown that metabolic disruption of astrocytes not only disrupts neuron‐glia energy coupling, but is also closely associated with a variety of neurodegenerative diseases and neuroinflammation, including MS, AD, PD, Huntington's disease (HD), and amyotrophic lateral sclerosis (ALS), among other disorders [9, 74]. This section systematically describes the metabolic regulatory network of astrocytes in physiological states. Taking AD as an example, the molecular mechanism of metabolic reprogramming during disease development is analyzed in depth.

### 3.1 Normal Metabolic Function of Astrocytes

**FIGURE 4:**
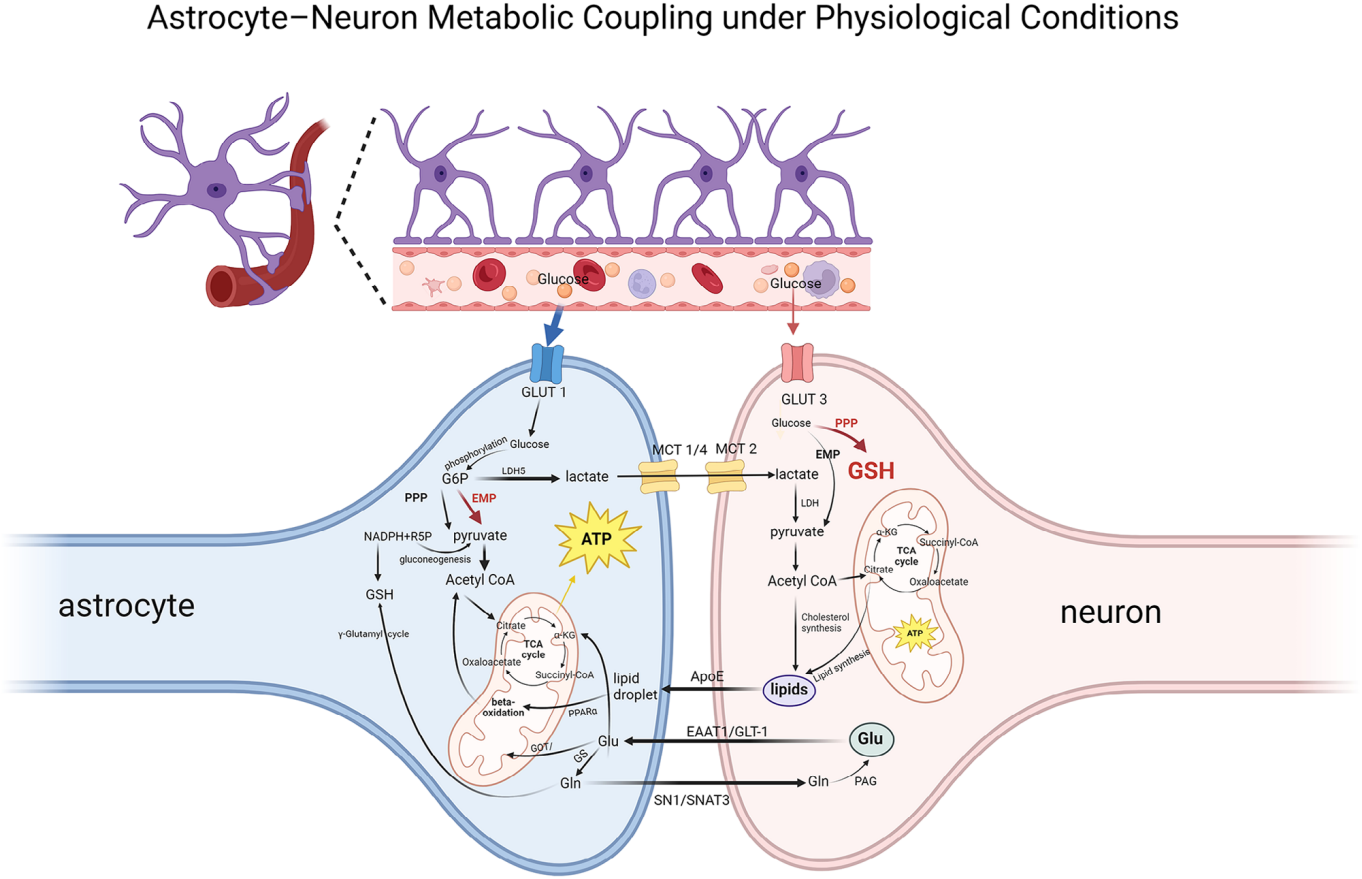
Astrocyte normal metabolism. After uptake of glucose by astrocytes, lactate is produced mainly by glycolysis and supplied to neurons as the main source of energy via the ANLS. Neurons are dependent on this because of their limited ability to produce their own energy. Lipotoxic substances produced by neuronal activity are transported to astrocytes for storage and oxidative detoxification; at the same time, glutamate released by neurons is taken up by astrocytes and recycled. Together, these synergistic mechanisms fulfil the core functions of energy supply, neuroprotection, and neurotransmitter recycling.

As the center of astrocyte metabolic network, glucose metabolism builds the molecular foundation of energy homeostasis in nerve cells. In the classical glucose metabolic pathway, glucose continuously outputs ATP required for maintaining neural homeostasis through the glycolytic pathway and the tricarboxylic acid cycle (TCA cycle), while biosynthetic precursors are exported by the pentose phosphate pathway (PPP). On the other hand, glucose metabolism provides neurons with lactate, which provides energy support for neuronal activity through the astrocyte‐neuron lactate shuttle model (ANLS) [6]. In addition, other metabolic pathways, such as lipid metabolism, and the glutamate–glutamine cycle are also important components of astrocyte energy metabolism. The normal metabolism of astrocytes is shown in Figure 4.

#### 3.1.1 Glucose Metabolism

The classical theory of glucose metabolism suggests that glucose crosses the BBB via the glucose transporters (GLUT) family [75]. In astrocytes, glucose molecules taken up by GLUT1 isoforms are phosphorylated by hexokinase to form glucose‐6‐phosphate (G6P), a step that constitutes the initiation of glucose metabolism. Subsequently, G6P has two major metabolic pathways. First, G6P is metabolized by the glycolysis pathway (Embden–Meyerhof–Parnas pathway) to form pyruvate, which is catalyzed by the pyruvate dehydrogenase complex to form acetyl‐CoA, and then enters the TCA cycle. This is followed by the TCA cycle, which ultimately produces adenosine triphosphate (ATP) via the oxidative phosphorylation pathway (OXPHOS), a common pathway for cellular energy production. Second, G6P can be metabolized via the PPP, where it oxidizes itself to produce ribulose‐5‐phosphate (R5P) with the synthesis of reducing equivalents of nicotinamide adenine dinucleotide phosphate (NADPH). R5P as a biosynthesis precursor R5P acts as a biosynthetic precursor in providing raw material for nucleotide synthesis, whereas NADPH has a key role as an intracellular reductant in antioxidant defense and biosynthesis. In addition, through the gluconeogenesis pathway, R5P can be isomerized to generate glyceraldehyde‐3‐phosphate and fructose‐6‐phosphate (F6P), which are catalyzed by hexose phosphate isomerase and can re‐enter the glycolytic pathway for cellular energy supply [76].

Of particular note is the existence of metabolic coupling between astrocytes and neurons—the ANLS [6]. Astrocytes produce lactate in large quantities through high expression of hexokinase Type II and lactate dehydrogenase isoenzyme 5, which are transported through the isoform‐specific transport of monocarboxylate transport proteins (monocarboxylate transporters [MCTs]). Astrocytes realize monocarboxylic acid transport and intercellular information exchange through MCT1‐CAII complex as input and MCT4‐PGK complex as output [77]. Meanwhile, MCT2 mediates neuronal uptake and transfers lactate to neuronal mitochondria to be converted to pyruvate by lactate dehydrogenase, which ultimately provides the energy base for neuronal activity through OXPHOS [78].

Recently, a number of teams have revealed new advances in the study of glucose metabolism in the nervous system, such as the molecular mechanisms involved in the regulation of glucose metabolism by the APC/C‐Cdh1 ubiquitin ligase system and in the synergy of glial cell‐neuron metabolism. The higher activity of neuronal APC/C‐Cdh1 sustains the degradation of the key regulatory enzyme of glycolysis, PFKFB3 (6‐phosphofructo‐2‐kinase/fructose‐2,6‐bisphosphatase isoform 3), through the ubiquitin‐proteasome system, resulting in a limited glycolytic capacity and inability to be upregulated under stress, which is physiologically coupled with its features of efficient assembly of the mitochondrial respiratory chain and low levels of mitochondrial ROS [79]. Metabolic reprogramming forces neurons to preferentially direct G6P to the PPP, which promotes the regeneration of reduced glutathione (GSH) to maintain redox homeostasis, but at the expense of glycolytic energy supply [80]. In contrast, APC/C‐Cdh1 activity is significantly suppressed in astrocytes, and PFKFB3 degradation is reduced, resulting in an adequate energy supply for glial cells [81]. Lactate is provided to neurons as an alternative energy substrate via the ANLS, while its own higher abundance of m ROS produced due to mitochondrial Complex I activates specific transcriptional programs to support neurocognitive function. Notably, the regulation of PFKFB3 is spatiotemporally specific: its abundance is controlled by a cascade of dual ubiquitination systems of APC/C‐Cdh1 (via the KEN box) and SCF‐β‐TrCP (via the DSG box) during cell cycle progression, which ensures that glycolysis is upregulated in response to nutrient signals at specific stages of G1 phase [82]. This mechanism not only explains the metabolic heterogeneity of neurons and glial cells, but also suggests the centrality of PFKFB3 as a metabolic checkpoint—its dynamic balance is directly related to cell proliferation, oxidative stress tolerance, and energy allocation strategies, which profoundly affects the physiological homeostasis and pathological adaptation of the nervous system.

#### 3.1.2 Lipid Metabolism

Astrocytes also play a key role in the regulation of lipid metabolism in the nervous system, and protect neurons from lipotoxic damage through dynamic lipid redistribution and metabolic detoxification mechanisms. It has been shown that excess free fatty acids (FAs) produced by neurons under high electrical activity can be transferred to astrocytes and stored in their intracellular lipid droplets (LDs) via the ApoE‐dependent lipid particle transport system [83, 84]. This metabolic coupling mechanism reveals the importance of the dynamic balance of lipid metabolism between neurons‐astrocytes in maintaining the functional integrity of neurons. Further studies revealed that astrocytes specifically express key components of the sterol regulatory element‐binding protein (SREBP) signaling pathway, including SREBP sheltering activation protein (SCAP) and its downstream target gene fatty acid synthase (FASN), while the pathway activity is significantly restricted in neurons [85, 86]. In a genetically engineered mouse model, astrocyte‐specific knockdown of SCAP leads to blockage of SREBP shear activation, which in turn inhibits cholesterol and phospholipid biosynthesis and secretion. Notably, the hippocampus of such mutant mice showed significant abnormalities in synaptic development: an increased proportion of immature synapses, downregulation of the expression of the presynaptic membrane marker protein SNAP‐25, and a reduced number of synaptic vesicles, accompanied by impaired long/short‐term synaptic plasticity [85]. These phenotypes suggest that lipid molecules synthesized by astrocytes through the SREBP‐FASN axis are indispensable for the regulation of structural maturation and functional plasticity of presynaptic terminals. This mechanism not only elucidates the non‐cell‐autonomous regulation of astrocyte lipid metabolism in synaptic development, but also provides a molecular explanation for the association between lipid metabolism disorders and synaptic dysfunction in neurodevelopmental and degenerative diseases.

#### 3.1.3 Amino Acid Metabolism

Glutamate, as the major excitatory neurotransmitter in the CNS, its precise regulation across cellular homeostasis is the core biological basis for maintaining normal brain function. Neurons and astrocytes achieve dynamic homeostasis of intersynaptic glutamate through a highly specialized metabolic coupling system, the glutamate–glutamine cycle [7]. Glutamate released by neurons is actively taken up by astrocytes via the high‐affinity sodium‐dependent glutamate transporters GLAST/EAAT1 and GLT1/EAAT2, and is subsequently converted to glutamine by the enzyme GS. Glutamine is retranslocated into neurons via specific transporters (SN1/SNAT3) and hydrolyzed and regenerated to glutamate by PAG, constituting a closed loop of the transmitter cycle [87]. This loop is not only closely linked to brain energy metabolism (ATP consumption during glutamate uptake in astrocytes drives sodium–potassium pumps to maintain the ionic gradient), but also integrates glutamate into the glucose and amino acid metabolism network through a metabolic shunt mechanism—glutamate in astrocytes can be hydrolyzed and regenerated into glutamate by either glutamate–asparagine aminotransferase or pyruvate carboxylase (PC)‐catalyzed entry into the TCA cycle, or via the γ‐glutamyl cycle for GSH biosynthesis, thus serving as a central hub linking carbon–nitrogen metabolism [88].

### 3.2 Metabolic Abnormalities of Astrocytes in Neurological Diseases

AD, one of the most common neurodegenerative diseases in the world, is characterized by pathological changes including senile plaques formed by Aβ deposition and neuroprogenitor fibrillar tangles caused by abnormal phosphorylation of tau protein [89]. Clinical and basic research evidence suggests that astrocytes are deeply involved in the pathological process of AD through dysregulation of the metabolic regulatory network: impaired glucose metabolism triggered by downregulation of the expression of the glucose transporter GLUT1 occurs in the early stages of the disease, leading to inadequate neuronal energy supply [90]. This is accompanied by an imbalance in lipid metabolism associated with the APOE4 genotype, which accelerates the abnormal deposition of LDs and neuroinflammatory responses [91]. Simultaneously, abnormal mitochondrial dynamics (fusion/disintegration imbalance) and impaired OXPHOS create irreversible neuronal degenerative changes [92]. In this section, the metabolic reprogramming features of astrocytes during AD progression will be analyzed in depth using AD as an example.

#### 3.2.1 Abnormalities of Glucose Metabolism

Disorders of brain glucose metabolism are central to the pathology and early drivers of AD. There is a significant temporal and spatial correlation between the pathology of AD and hypometabolism, which is not only an early biomarker of AD, but also a key mechanism driving neurodegeneration. Studies have shown that reduced GLUT1 expression worsens cerebrovascular degeneration, neuropathology, and cognitive function in AD [90]. Kyrtata et al. found that GLUT1 and GLUT3 were reduced in hippocampal and cortical regions of AD patients and were hypothesized to be caused by high levels of Aβ. Interestingly, levels of GLUT2 and GLUT12 were increased, possibly due to compensatory effects [93].

In addition, downregulation of the expression of the key transporter of the ANLS, MCT1/4, reduced neuronal lactate supply by 60%. This metabolic disorder exacerbates the pathological process through a multidimensional cascade of reactions: (1) impaired mitochondrial OXPHOS leads to reduced ATP synthesis and neuronal energy crisis triggering calcium homeostasis imbalance and synaptic dysfunction; (2) compensatory enhancement of glycolysis triggers lactate accumulation, which promotes pro‐inflammatory gene expression through epigenetic modifications (e.g., histone lactylation); and (3) reduced flux of the PPP, resulting in inadequate NADPH production, weakens the antioxidant defense system and exacerbates oxidative stress. Notably, decreased activity of thiamine (vitamin B1)‐dependent enzymes (e.g., pyruvate dehydrogenase complex and transketolase) further disrupts the TCA cycle and neurotransmitter synthesis, creating a metabolic‐oxidative vicious cycle [94].

#### 3.2.2 Lipid Metabolism Disorders and Abnormalities of Mitochondrial Metabolism

Abnormal metabolism in astrocytes plays a key role in neurodegenerative diseases such as AD, and the central mechanism is closely related to lipid metabolism disorders driven by APOE4 genotypes. Homozygous and heterozygous carriers of APOE4 have a 12‐fold and 2–3‐fold increased risk of late‐stage AD, respectively, compared to carriers of APOE2 or APOE3 [95]. APOE4‐expressing astrocytes exhibited altered synthesis of mitochondrial kinetic proteins, ubiquitination, and proteasome/lysosome degradation, as evidenced by more mitochondrial fusion and less mitochondrial autophagy. At the same time, cellular mitochondrial function was impaired, as evidenced by lower mitochondrial cristae density and reduced intracellular ATP levels [96]. Studies have shown that astrocytes carrying APOE4 exhibit a significant imbalance in the dynamics of LDs: compared to the APOE3 genotype, APOE4 astrocytes have an increased number of LDs but a reduced size, accompanied by an upregulation of the expression of the droplet‐stabilizing protein perilipin‐2, suggesting impaired turnover of the droplets and the accumulation of toxic metabolites [97]. LD accumulation has a dual nature: short‐term protection of cells, but long‐term dysregulation drives chronic cellular stress and forms the basis of AD pathology [98]. Decreased LD mobilization/scavenging and impaired mitochondrial β‐oxidation by ApoE4 astrocytes lead to the accumulation of incomplete oxidation intermediates (e.g., acylcarnitines), along with compensatory enhancement of endogenous FA oxidation (elevated rate of oxygen consumption) but ultimately exacerbate oxidative stress and energy crisis due to inhibition of mitochondrial function [99]. In addition, APOE4 exacerbates the pathological process by disrupting neuronal‐astrocyte metabolic collaboration: reduced segregation of FA by LDs within neurons leads to toxic FA accumulation, while APOE4‐dependent decreased transport efficiency of ApoE‐positive lipid particles further blocks astrocyte reception and detoxification of neuron‐derived FA [98, 99]. Inhibition of stearoyl‐CoA desaturase‐1 (SCD‐1) or choline supplementation reverses lipid accumulation in E4 cells, suggesting that an imbalance in monounsaturated fatty acid synthesis is a central mechanism of ApoE4 astrocytes lipid metabolism disorders [100]. Study shows that SCD‐1 levels are elevated in the brains of AD patients and that inhibition of SCD‐1 ameliorates cognitive deficits in an AD mouse model [101, 102].

Endocytosis and lysosomal dysfunction also play an important role in lipid metabolism disorders. Neurons expressing ApoE4 exhibit disrupted endolysosomal trafficking, characterized by enlarged lysosomes and accumulation of Rab5a‐positive early endosomes, indicating impaired vesicular transport and degradation pathways [103, 104]. Primary mouse neurons treated with E4 interfere with ApoE R2 recycling, decrease neuronal sensitivity to reelin and NDMA and AMPA receptors, and impair synaptic plasticity [105]. APOE4 astrocytes have reduced lysosomal pH, decreased low‐density lipoprotein receptor‐related protein 1 (LRP1) surface expression, and decreased endocytosis (e.g., reduced epidermal growth factor and transferrin uptake) [106, 107]. This transcellular metabolic uncoupling not only drives AD pathology (e.g., Aβ deposition and tau hyperphosphorylation), but also creates a “metabolic‐inflammatory vicious cycle” by activating neuroinflammation [108]. The above findings reveal the centrality of astrocyte lipid metabolism abnormalities in neurodegenerative diseases, and provide key therapeutic directions for targeting APOE4, regulation of LD dynamics and transcellular metabolic remodeling.

#### 3.2.3 Glutamate Cycle Imbalance

As mentioned earlier, L‐glutamate as a core excitatory neurotransmitter in the CNS, dynamically regulates synaptic plasticity, learning memory, and neural circuit integration through activation of ionotropic (e.g., NMDA and AMPA receptors) and metabotropic glutamate receptors (mGluRs) [109]. However, precise spatiotemporal regulation of their extracellular concentrations is critical for neuronal survival. Glutamate transporter proteins, also known as EAATs, maintain extracellular glutamate concentrations at submicromolar levels by rapidly removing glutamate released from synaptic gaps through a high‐affinity, sodium‐dependent uptake system (with peak concentrations up to the millimolar level). This process is dependent on an electrochemical gradient drive and involves the isotransport of 3 Na⁺ and 1 H⁺ and the reverse transport of 1 K⁺ [110]. If transport is dysfunctional (e.g., reduced EAAT expression or inhibition of activity), glutamate overaccumulation can trigger receptor overactivation, leading to neuroinflammation, mitochondrial dysfunction, and oxidative stress cascades, ultimately triggering excitotoxicity—a mechanism that has been implicated in epilepsy, PD, ALS, and AD, among other neurodegenerative diseases [111, 112, 113].

The typical pathological features of AD‐Aβ deposition, tau protein hyperphosphorylation, and synaptic loss are profoundly linked to imbalances in glutamatergic homeostasis. GLT‐1/EAAT‐2 expression is significantly reduced in the brains of patients with AD and the extent of its deficiency is positively correlated with cognitive decline [114, 115]. Scimemi et al. found that Aβ42 exacerbates glutamatergic homeostatic imbalance by interfering with the subcellular localization and function of GLT‐1 [116]. Animal models further demonstrated that early genetic reduction of GLT‐1 in mice accelerates amyloid plaque deposition and cognitive decline; pharmacological upregulation of GLT‐1 showed neuroprotective effects [114]. Progerin 1 (PS1) is the catalytic component of γ‐secretase, which catalyzes the intramembrane cleavage of amyloid precursor protein (APP) to generate Aβ peptides of varying lengths [117]. Most familial AD‐associated PS1 mutations directly promote amyloid plaque formation by increasing the production of neurotoxic Aβ (e.g., Aβ42). PS1 binds directly to GLT‐1 in neurons and astrocytes, whereas the PS1 D257A mutation and the γ‐secretase inhibitor DAPT do not affect PS1 binding to GLT‐1, suggesting that the two interact independently of γ‐secretase enzyme activity [114]. Although it is controversial whether GLT‐1/EAAT‐2 downregulation is the initiator of AD, its role as an amplifier of disease progression is widely supported, and targeting to enhance its function or reverse aberrant localization has emerged as a potential therapeutic strategy.

## 4 Therapeutic Approaches Targeting Astrocytes in Neurological Diseases

**FIGURE 5:**
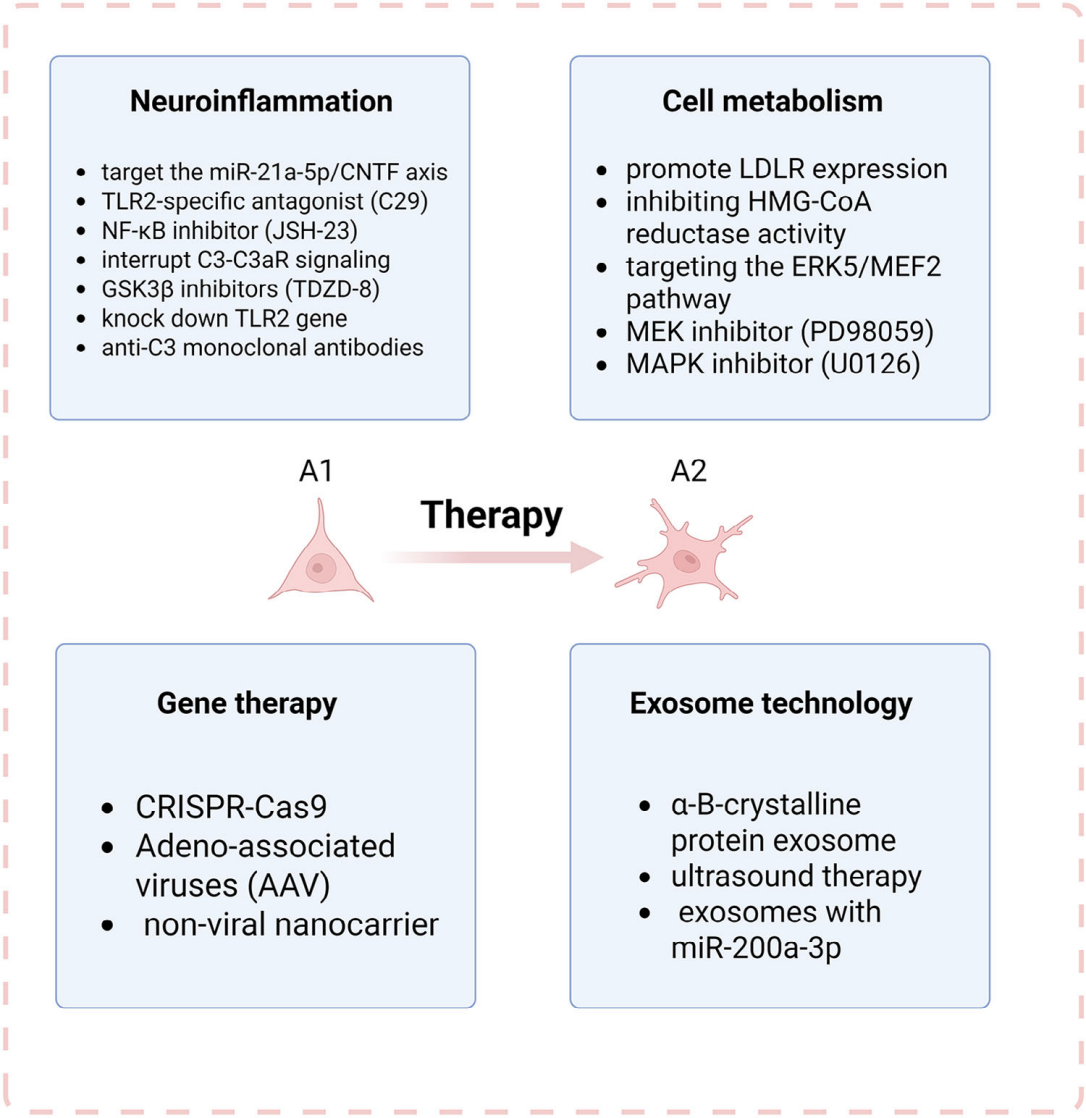
Therapeutic approaches targeting astrocytes in neurological diseases. Neuroinflammation targets include modulating astrocyte polarization, blocking pro‐inflammatory signals, and inhibiting complement‐mediated toxicity. Metabolic regulation focuses on lipid homeostasis and its interaction with inflammation. Cutting‐edge delivery approaches involve gene therapy and exosome technology.

Astrocytes have become a key target for the treatment of neurological disorders due to their important role in the nervous system. In recent years, breakthroughs in multi‐omics technologies (e.g., transcriptomics, proteomics, and metabolomics), combined with innovative clinical trial designs, have significantly advanced the development of therapeutic approaches targeting astrocytes. In this section, based on the latest research progress and clinical trial data registered by the National Institutes of Health (NIH), we systematically review the current research on astrocyte‐targeted neurological diseases, with a focus on the mechanism innovation and clinical translation potential (Figure 5).

### 4.1 Modulating Astrocyte Activation and Neuroinflammation

Astrocytes exhibit remarkable heterogeneity in neurological diseases, in which the dynamic balance between the pro‐inflammatory A1 subtype and the neuroprotective A2 subtype directly determines disease progression. A1 astrocytes exacerbate neuroinflammation through the secretion of pro‐inflammatory factors (e.g., IL‐1α and TNF‐α) and complement proteins (e.g., C‐1q), which lead to synaptic loss and myelin damage to the point of neuronal death. In contrast, the A2 subtype promotes repair by releasing anti‐inflammatory factors (e.g., IL‐1β) and neurotrophic factors (e.g., Nuclear factor IA) [74]. Therefore, modulating the polarization state of astrocytes has become a central direction for therapeutic strategies.

Clinical interventions can be targeted at the inhibition of inflammatory signaling pathways. For example, inhibition of STAT3 phosphorylation significantly reduces the release of pro‐inflammatory factors through the NF‐κB/STAT3 pathway, a central driver of A1 astrocyte activation [118, 119]. Zhang et al. found that miR‐21a‐5p promotes inflammation through upregulation of A1 polarization by inhibiting the CNTF/STAT3/Nkrf pathway. Evidence suggested that loss‐of‐function of miR‐21a‐5p significantly enhances the inhibitory effect of CNTF on A1s, whereas Cntfr α knockdown completely reverses this effect. In an animal model of traumatic spinal cord injury (TSCI), miR‐21a‐5p inhibitor significantly reduced the proportion of A1s and improved nerve regeneration by enhancing CNTF/STAT3 signaling axis activity, a neuroprotective effect that was lost in astrocyte‐specific STAT3‐deficient mice. Notably, even without administration of exogenous CNTF, miR‐21a‐5p itself could slightly affect the A1/A2 marker balance by regulating the basal expression level of Cntfr α, suggesting a ligand‐independent basal regulatory function [119]. Therefore, targeting the miR‐21a‐5p/CNTF axis to treat neurodegenerative diseases remains to be realized in the future.

Chi et al. found that α‐synuclein (α‐syn) significantly upregulated complement C3 secretion by activating astrocyte TLR2 receptors and triggering the NF‐κB signaling pathway (inhibition of TLR2/NF‐κB resulted in a significant decrease in fluorescence intensity). Astrocyte‐derived C3 exacerbates α‐syn PFF‐induced neuronal apoptosis upon binding to C3aR [120]. Therefore, based on the mechanisms described above, therapeutic strategies could be to block C3 production at the source, for example, by applying a TLR2‐specific antagonist (e.g., C29) or an NF‐κB inhibitor (JSH‐23), and to interrupt C3‐C3aR signaling, for example, by using a C3aR antagonist (SB290157) treatment attenuates tau hyperphosphorylation through the GSK3β signaling pathway and inhibits neuronal apoptosis. Treatment with GSK3β inhibitors (e.g., TDZD‐8) can also reduce primary neuronal apoptosis induced by complement C3 protein and α‐syn PFF. In addition, future studies are pending to employ gene editing techniques to specifically knock down the astrocyte *TLR2* gene or use anti‐C3 monoclonal antibodies to reduce α‐syn pathological load and inhibit neuronal apoptosis.

In AD, NF‐E2‐related factor 2 (Nrf2) blocks A1‐type astrocyte formation through inhibition of NF‐κB subunit p65 recruitment and regulates neuroinflammation through C3‐STAT3 signaling [121, 122]. PD pathology involves contrasting regulatory mechanisms: RGS5 aggravates neurodegenerative pathology by enhancing TNFR signaling, whereas NR1H4 confers neuroprotection through CEBPβ/NF‐κB pathway inhibition [123, 124]. In epilepsy models, SerpinA3N exacerbated KA‐induced neuroinflammation by activating NF‐κB signaling and correlated with RYR2 phosphorylation. In ischemic stroke pathology, lipocalin‐2 (LCN2) mediated NLRP3 inflammatory vesicle‐dependent astrocyte death via the 24p3R receptor, whereas ethanol activated NLRP6 inflammatory vesicles via miR‐339 downregulation [125, 126, 127]. In methamphetamine‐induced neuroinflammation, IL‐10 and Cdc42 regulate astrocyte‐microglia interactions via Rho GTPase [128]. The LR agonist PEDF‐34 inhibits astrocyte A1 polarization via the JNK/STAT1 pathway and ameliorates neuroinflammation in subarachnoid hemorrhage, whereas deletion of Nurr1 improves neuroinflammation by decreasing GDNF, increasing proinflammatory factor release and disrupting BBB integrity [129, 130]. Collectively, these investigations establish astrocytes as central orchestrators of neuroinflammation and cell death through intricate, disease‐specific molecular networks, thereby identifying critical intervention nodes for the development of precision therapeutic strategies targeting astrocytic dysfunction.

### 4.2 Metabolic Interventions to Restore Astrocyte Function

As a hub of metabolic regulation in the CNS, the dysfunction of astrocytes is closely related to neuroinflammatory and degenerative diseases. In recent years, significant breakthroughs have been made in the treatment of neurological diseases by restoring astrocyte function, and multi‐omics technologies have provided new perspectives for analyzing their pathological mechanisms and developing precise therapeutic strategies.

Restoring mitochondrial OXPHOS in astrocytes. Studies have shown that mitochondrial metabolic disorders in astrocytes of AD patients are manifested by abnormally elevated activity of pyruvate dehydrogenase kinase (PDK), leading to an imbalance in glycolysis/OXPHOS [131]. Recently, Khan et al. found that an essential molecule called PDK inhibitor dichloroacetate (DCA) augments usage of the glycolysis‐produced pyruvate in the mitochondria increasing OXPHOS [132]. To support this metabolic switch, DCA‐treated cells significantly upregulate low‐density lipoprotein receptor (LDLR) expression, promoting exogenous cholesterol and triglyceride uptake. ERK5 kinase is a key regulator of metabolic substrate selection, and the use of ERK5 inhibitors (e.g., XMD8‐92) results in a reduction of LDLR protein. MEF2 family transcription factors directly bind to the LDLR promoter (ChIP‐seq validation of MEF2 binding sites) and mediate the regulation of LDLR by ERK5. ERK5 forms the “ERK5‐MEF2‐LDLR” transcriptional complex through phosphorylation of MEF2A/C/D (serine/threonine sites) and direct coactivation [132]. Therefore, future treatment is expected to promote LDLR expression indirectly by inhibiting HMG‐CoA reductase activity through DCA, which increases the cellular demand for exogenous cholesterol. Meanwhile, targeting the ERK5/MEF2 pathway to develop specific activators, such as berberine to induce LDLR expression through the ERK5‐dependent pathway and to circumvent AMPK‐dependent side effects, or designing BBB‐impenetrable DCA derivatives; PD98059 (MEK inhibitor) and U0126 (MAPK inhibitor) blocked DCA‐induced LDLR effects, suggesting that critical status of the ERK5 pathway, providing a basis for the development of novel lipid‐lowering combination therapies.

The emerging paradigm of astrocytic metabolic reprogramming and inflammatory regulation represents a critical frontier in understanding brain disease pathogenesis. In cerebral ischemia models, lactate functions as a neuroprotective metabolite by inhibiting oxygen glucose deprivation (OGD)‐induced TNF‐α expression through NDRG2 protein stabilization, thereby exerting potent anti‐inflammatory effects that mitigate ischemic injury [133]. Similarly, in neonatal hypoxic‐ischemic encephalopathy (HIE), 4‐octyl itaconic acid ester (4OI) orchestrates astrocytic neuroprotection by activating the Nrf2 pathway to synergistically modulate oxidative stress and inflammatory responses [134]. Advanced metabolic imaging of AD patients reveals distinctive regional patterns: the olfactory entorhinal cortex, hippocampus, and temporoparietal regions exhibit a characteristic metabolic dissociation between elevated 11C‐acetate uptake and diminished 18F‐FDG metabolism. Importantly, 11C‐labeled astrocytic functional decline in carriers of disease‐causing mutations correlates significantly with 18F‐labeled progressive hypometabolism, establishing a valuable biomarker for dynamic monitoring of AD progression [135]. In TBI paradigms, sodium pyruvate, ethylpyruvate, and glucose demonstrate differential efficacy in restoring neuron‐glial metabolic coupling following controlled cortical injury through enhanced lactate production and restoration of PC activity [136]. Conversely, pathological metabolic disruption is exemplified by chronic intermittent ethanol exposure, which induces persistent impairment of central lactate transport through MCT upregulation, revealing a key metabolic mechanism underlying alcohol‐related neurological injury [137]. From a therapeutic perspective, ginsenoside Rb1 demonstrates remarkable neuroprotective efficacy by attenuating astrocyte activation and mitochondrial dysfunction after ischemic stroke through targeted inhibition of NADH dehydrogenase activity in mitochondrial Complex I and suppression of reverse electron transfer‐mediated ROS generation [138]. Collectively, these investigations illuminate the sophisticated multidimensional mechanisms through which astrocytes orchestrate neuropathological processes via the metabolic‐inflammatory axis, establishing a compelling foundation for developing precision neuroprotective strategies based on metabolic intervention.

### 4.3 Gene Therapy Approaches

Gene therapy provides a strategy for root cause intervention in neurodegenerative diseases by correcting aberrant gene expression or repairing disease‐causing mutations in astrocytes. Based on technological advances in viral vectors, gene editing, and synthetic biology, current research focuses on the regulation of key metabolic pathways, correction of disease‐causing genes, and development of precision delivery systems.

In early‐onset AD, mutants PSEN1 and PSEN2 accelerate Aβ1‐42 production by altering the APP cleavage site. And using CRISPR‐Cas9 can correct the PSEN2 N141I mutation in patient induced pluripotent stem cells (iPSCs), restoring the Aβ42/40 ratio to normal levels and reversing the electrophysiological defects in neurons [139]. By targeting the C‐terminal structural domain of the *APP* gene, CRISPR technology blocked its interaction with β‐secretase (BACE1) and significantly reduced Aβ formation [140].

The use of viral vectors to deliver CRISPR/Cas9 is by far the most widely used and classical method, though it may have significant adverse effects due to off‐target mutations. Adeno‐associated viruses (AAV) are widely used for delivery, but their packaging capacity is limited (< 4.8 kb) and they may trigger an immune response [141]. For example, György et al. used a dual AAV vector partitioning strategy to load the sgRNA and Cas9 coding sequences targeting the APPSW mutant allele into AAV vectors separately. The in vitro model isolated primary neurons from APPSW transgenic Tg2576 mouse embryos for injection of dual AAV vectors, and in vivo validation by localized injection of dual AAV vectors into the hippocampal region of adult Tg2576 mice. The results of both the in vitro and in vivo experiments showed that CRISPR/Cas9 can selectively disrupt APP and thus reduce pathogenic Aβ [142].

In addition, rapid progress has been made in nonviral nanocarrier technology, which offers lower cost, higher specificity, higher sample load, and lower immunotoxicity [143]. Park et al. used a Cas9‐sgRNA ribonucleoprotein that specifically targets BACE1, which forms a complex with a nanocomplex made of R7L10 peptide. The results showed that the complex did not have any substantial off‐target mutations in vivo and reduced BACE1 expression, inhibiting Aβ‐related pathology and cognitive deficits in two mouse models of AD [144]. Non‐viral vectors, despite having lower off‐target risk and delivery efficiency than viral vectors, still have challenges such as poor penetration and vector material‐related toxicity. In the future, the combination of gene editing and epigenetic modulation technology is needed to further promote the translation of clinical diseases.

Recent breakthroughs in astrocyte‐targeted gene therapy have established innovative strategies for neurodegenerative diseases and brain injury repair. In the treatment of ischemic brain injury, NeuroD1 AAV gene therapy effectively repairs neuronal functional deficits by converting glial cells into functional new neurons through in vivo cellular reprogramming technology [145]. For dopaminergic neurodegeneration in PD, IGF‐1 gene therapy not only significantly improves cognitive functions, such as short‐term memory and spatial working memory, but also upregulates the expression of tyrosine hydroxylase in the caudate‐crustal nucleus (CPu), and remodels the function of nigrostriatal pathway [146]. In the therapeutic field of HD, a two‐pronged strategy has achieved important breakthroughs: on one hand, AAV‐mediated co‐expression of NeuroD1 and Dlx2 transcription factors reprogrammed striatal astrocytes into GABAergic neurons, significantly extending the lifespan of the R6/2 model mice and improving motor function [147]; on the other hand, AAV2/5 vector‐driven SREBP2 overexpression significantly extended the lifespan of the mice by activating the cholesterol biosynthesis pathway [148]. Expression improved the HD pathological phenotype multidimensionally by activating the cholesterol biosynthesis pathway, restoring the synaptic transmission function, reversing the aberrant expression of dopamine receptor D2 (Drd2), and removing the mutant Huntington's protein aggregates. For vanishing white matter disease (VWM), characterized by white matter loss, the AAV9‐gfaABC(1)D‐EIF2B5 vector achieved astrocyte‐specific gene delivery through the GFAP promoter, demonstrating that targeting and correcting astrocyte dysfunction represents a critical component of disease treatment [149]. These studies not only demonstrate the unique plasticity of astrocytes as prime targets for gene therapy, but also establish a comprehensive multi‐level therapeutic framework spanning cellular transformation to molecular repair through precise regulation of cholesterol metabolism, synaptic plasticity, and protein homeostasis pathways, thereby laying robust theoretical and practical foundations for clinical translation.

### 4.4 Novel Strategies Utilizing Astrocyte‐Derived Exosomes and Nanotechnology

Exosomes are a type of extracellular vesicle and can be produced by almost all eukaryotic cells. In the CNS, neurons, microglia, astrocytes, oligodendrocytes, and neural stem cells (NSCs) all secrete exosomes. Astrocytes, as abundant glial cells, also secrete exosomes with various components under different conditions, and can also receive exosomes from other cells to change their functions and phenotypes [150]. Therefore, exosome therapy targeting astrocytes has gained the attention of many researchers.

Exosomes have a bidirectional signaling role. Exosomes secreted by other cells regulate astrocyte function by delivering miRNAs, proteins, and other molecules. For example, mesenchymal stem cell (MSC)‐derived exosomes (MSC‐Exos) carry miR‐133b, which enhances neuroprotection in astrocytes [151]. In contrast, astrocytes secrete exosomes (AS‐Exos) carrying apolipoprotein D (ApoD), miRNAs (e.g., miR‐17‐5p), and so on, which affect neuronal survival, microglia polarization, and oligodendrocyte differentiation. AS‐Exos inhibits microglia activation and attenuates neuroinflammation through the expression of α‐B‐crystalline protein, whereas exosomes secreted by reactive astrocytes may carry pro‐inflammatory factors (e.g., IL‐6) or toxic proteins (e.g., lipocalin‐2) that exacerbate neuronal injury [152, 153].

Therapeutically, stimulation of astrocytes by ultrasound increases protective exosome release, which can attenuate Aβ toxicity to neurons in vitro; and when combined with focused ultrasound (FUS)‐induced opening of the BBB, it is able to clear Aβ in vivo [154]. In PD, AS‐Exos released exosomes with miR‐200a‐3p to neurons, targeting MKK4 by binding to two independent sites on the 3'‐UTR of Map2k4/MKK4 mRNA, attenuating cell death in MPP‐treated SH‐SY5Y cells and glutamate‐treated hippocampal neuronal cultures [155]. Astrocytes expressing mutant SOD1 activate unconventional secretory pathways that help to limit intracellular aggregate formation and overcome mutant SOD1 toxicity, with potential implications in the mechanisms of ALS development [156]. The core advantage of exosome therapeutic strategies lies in their excellent targeting and penetration properties, naturally carrying tissue‐specific surface proteins that can penetrate the BBB for CNS‐targeted delivery. Engineering modified exosomes (e.g., loading miRNA or CRISPR components) can precisely modulate astrocyte phenotype. Compared to viral vectors, exosomes (especially autologous sources) are less immunogenic, reducing therapeutic side effects.

As an important mediator of intercellular communication, exosomes exhibit multidimensional mechanisms of action in the pathological regulation and treatment of neurological diseases. MSC‐Exo ameliorated inflammation‐induced astrocyte dysfunction by activating Nrf2 and inhibiting the NF‐κB signaling pathway, significantly alleviating the neuroinflammatory and activation phenotypes in a mouse model, suggesting its therapeutic potential in neurodegenerative diseases [157]. In TBI, miR‐873a‐5p‐rich exosomes released from astrocytes targeted microglia and blocked LPS‐induced M1‐type polarization and pro‐inflammatory factor release by inhibiting ERK and NF‐κB p65 phosphorylation, while another astrocyte‐derived exosome, lncRNA 4933431K23Rik, blocked M1‐type polarization and pro‐inflammatory factor release by upregulating the physical binding of Smad7 to IκBα and inhibiting its ubiquitination degradation, which in turn blocked the overactivation of the NF‐κB signaling pathway, forming a dual anti‐inflammatory regulatory network [158, 159]. For ischemic stroke, AS‐Exo enhanced neuronal viability and inhibited apoptosis by decreasing the levels of caspase‐3, Bax, and pro‐inflammatory factors (TNF‐α, IL‐6, and IL‐1β); whereas, in acute ischemic stroke model, oxygen‐glucose deprivation/reperfusion (OGD/R)‐treated AS‐Exos (ADEXs) inhibited apoptosis by releasing nicotinamide phosphoribosyltransferase (Nampt), which activates the AMPK/mTOR pathway to induce autophagy, thereby attenuating neuronal damage [160]. In AD treatment, bone marrow BMSC‐exos significantly reduced the levels of Aβ1‐42, phosphorylated tau, and pro‐inflammatory factors (IL‐1β, IL‐6, and TNF‐α) by lateral ventricle injection, while upregulating synaptic proteins and BDNF expression to achieve the dual effects of neuroprotection and synaptic remodeling [161, 162]. Notably, exosomes in the pathological microenvironment may exhibit bidirectional effects: M1 microglia‐derived exosomes (M1‐exos) drive A1‐type astrocyte activation via the circSTRN3/miR331‐5p/MAVS/NF‐κB axis, exacerbating the inflammatory cascade response to ischemic brain injury [163]. These studies systematically reveal the core mechanisms by which exosomes precisely regulate neuroinflammation, metabolic reprogramming, and cell fate through the delivery of functional molecules (e.g., miRNAs, lncRNAs, and enzyme proteins), and provide theoretical rationale and translational direction for the development of exosome‐based targeted intervention strategies.

Overall, therapeutic innovations targeting astrocytes are rapidly advancing, with multi‐omics technologies enabling precise molecular localization while clinical trials validate translational potential. Future advances in CRISPR, nanotechnology, and artificial intelligence‐driven drug design are yet to be utilized to unlock the full therapeutic potential of astrocytes. By addressing current limitations and adopting emerging technologies, astrocyte‐targeted therapies may soon transition from the laboratory to the clinic, offering hope to patients with neurological disorders.

## 5 Future Directions and Perspectives

Astrocytes exhibit remarkable functional diversity in the context of CNS pathology, with their roles spanning a spectrum from neuroprotective to neurotoxic depending on the specific disease context, microenvironmental cues, and temporal dynamics of the pathological process. In CNS diseases, astrocytes show a complex dual role: they can attenuate inflammation and protect neurons through repair, but they can also exacerbate inflammation by releasing pro‐inflammatory factors. These cells function not only as architects of synaptic connectivity but also as critical regulators of neurotransmitter homeostasis, with their dysfunction representing a convergence point in the pathogenesis of various neurodegenerative conditions, including AD, PD, and ALS. Moreover, their role in maintaining BBB means that their dysfunction can increase permeability and accelerate disease progression. The therapeutic potential of targeting astrocyte function has garnered significant attention, with promising avenues including small molecule modulators of astrocyte reactivity, gene therapy approaches to restore beneficial astrocytic functions, and innovative nanotechnology‐based delivery systems for astrocyte‐specific interventions

**FIGURE 6:**
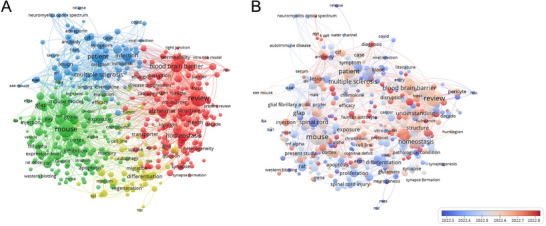
Bibliometric analysis based on articles around astrocytes and CNS in last 5 years. (A) Cluster analysis; (B) Time analysis.

To gain a comprehensive understanding of the evolving landscape of astrocyte research in neurological disorders, we conducted a bibliometric analysis of the scientific literature published within the past 5 years, employing network visualization techniques to identify key research clusters and temporal trends. The research focuses on four distinct groups of keywords (Figure 6A). The red cluster demonstrates strong associations with AD, BBB integrity, and astrocyte structural alterations, highlighting the convergence of research interest on astrocytic contributions to neurodegenerative pathophysiology and cerebrovascular dysfunction. The green cluster deals with mouse models, gene expression, and inflammation, indicating substantial investment in preclinical research methodologies that leverage animal models and molecular approaches to elucidate fundamental mechanisms underlying astrocyte function and neuroinflammatory responses. The third major cluster (blue) encompasses clinical dimensions including patient populations, symptomatology, and MS, reflecting the progressive translation of astrocyte research toward human disease applications, particularly in the context of autoimmune and inflammatory demyelinating disorders. The yellow cluster revolves around regeneration and differentiation, underscoring emerging interest in the therapeutic potential of manipulating astrocyte phenotypes to promote neural repair and functional recovery following CNS injury. Temporal analysis of research trends (Figure 6B) reveals a notable evolution in the focus of astrocyte research over recent years, with an increasing emphasis on their role in neurodegenerative diseases, particularly regarding BBB function and AD pathology. The color transition from blue to red indicates a growing emphasis on the permeability of BBB, neuroinflammation, and their role in disease progression. This progression illustrates a significant paradigm shift in our conceptualization of astrocytes—from traditional supporting cells to active participants in pathological processes and potential therapeutic targets. Furthermore, the research focus has shifted from basic animal model studies to more clinically relevant research, with increasing emphasis on approaches that evaluate astrocyte dysfunction in patient‐derived samples, neuroimaging correlates of astrocyte reactivity, and early‐phase clinical trials targeting astrocyte‐mediated pathways.

Despite the considerable promise of astrocyte‐directed therapeutic strategies, significant challenges persist in their clinical translation, necessitating further fundamental and applied research. While astrocyte‐targeted therapies hold great promise, translating these approaches into clinical practice remains challenging. Critical barriers include achieving sufficient cell‐type specificity to avoid unintended effects on non‐astrocytic cell populations, developing robust biomarkers to monitor astrocyte reactivity states in vivo, and determining optimal therapeutic windows for intervention, as the functional consequences of astrocyte modulation may vary dramatically depending on the disease stage. From improving specificity to overcoming side effects, future research must refine these techniques to develop safer and more effective treatments. The heterogeneity of astrocyte populations across different brain regions and their disease‐specific responses further complicates therapeutic development, suggesting that successful treatment approaches may require region‐specific and context‐dependent targeting strategies. Perhaps most promisingly, the emerging concept of personalized medicine approaches targeting astrocytes represents a frontier with significant therapeutic potential. In addition, personalized treatment strategies based on the reactive states of individual astrocytes may become a major breakthrough in the future of medicine. By integrating multi‐omics profiling of patient‐derived astrocytes, advanced neuroimaging techniques that visualize specific aspects of astrocyte function, and precision pharmacology approaches, it may become possible to stratify patients based on their astrocytic phenotypes and tailor therapeutic interventions accordingly. Such precision medicine approaches could dramatically improve treatment outcomes by addressing the substantial heterogeneity observed in neurological disorders and acknowledging the complex, multifaceted roles of astrocytes in health and disease.

## Author Contributions

M.Z.: conceptualization, writing – review and editing, supervision, funding acquisition, project administration. M.H., A.L., L.H.: data curation, methodology, writing – original draft, validation. Z.S.: visualization, editing. All authors have read and approved the final manuscript.

## Ethics Statement

The authors have nothing to report.

## Conflicts of Interest

The authors declare no conflicts of interest.

## Data Availability

Data availability is not applicable to this article as no new data were created or analyzed in this study.
